# Integrated genetic analysis of leaf blast resistance in upland rice: QTL mapping, bulked segregant analysis and transcriptome sequencing

**DOI:** 10.1093/aobpla/plac047

**Published:** 2022-10-14

**Authors:** Qingqun Tan, Haiyong He, Wen Chen, Lu Huang, Dailin Zhao, Xiaojun Chen, Jiye Li, Xuehui Yang

**Affiliations:** Guizhou Institute of Plant Protection, Guizhou Academy of Agricultural Sciences, Guiyang 550009, China; Guizhou Institute of Plant Protection, Guizhou Academy of Agricultural Sciences, Guiyang 550009, China; Guizhou Institute of Plant Protection, Guizhou Academy of Agricultural Sciences, Guiyang 550009, China; Guizhou Institute of Plant Protection, Guizhou Academy of Agricultural Sciences, Guiyang 550009, China; Guizhou Institute of Plant Protection, Guizhou Academy of Agricultural Sciences, Guiyang 550009, China; Guizhou Institute of Plant Protection, Guizhou Academy of Agricultural Sciences, Guiyang 550009, China; Guizhou Institute of Plant Protection, Guizhou Academy of Agricultural Sciences, Guiyang 550009, China; Guizhou Institute of Plant Protection, Guizhou Academy of Agricultural Sciences, Guiyang 550009, China

**Keywords:** Blast resistance, BSA, bulk segregant analysis, mapping, QTL, resistance breeding, transcriptome, upland rice

## Abstract

Elite upland rice cultivars have the advantages of less water requirement along with high yield but are usually susceptible to various diseases. Rice blast caused by *Magnaporthe oryzae* is the most devastating disease in rice. Identification of new sources of resistance and the introgression of major resistance genes into elite cultivars are required for sustainable rice production. In this study, an upland rice genotype UR0803 was considered an emerging source of blast resistance. An F_2_ mapping population was developed from a cross between UR0803 and a local susceptible cultivar Lijiang Xintuan Heigu. The individuals from the F_2_ population were evaluated for leaf blast resistance in three trials 7 days after inoculation. Bulked segregant analysis (BSA) by high-throughput sequencing and SNP-index algorithm was performed to map the candidate region related to disease resistance trait. A major quantitative trait locus (QTL) for leaf blast resistance was identified on chromosome 11 in an interval of 1.61-Mb genomic region. The candidate region was further shortened to a 108.9-kb genomic region by genotyping the 955 individuals with 14 SNP markers. Transcriptome analysis was further performed between the resistant and susceptible parents, yielding a total of 5044 differentially expressed genes (DEGs). There were four DEGs in the candidate QTL region, of which, two (*Os11g0700900* and *Os11g0704000*) were upregulated and the remaining (*Os11g0702400* and *Os11g0703600*) were downregulated in the susceptible parent after inoculation. These novel candidate genes were functionally annotated to catalytic response against disease stimulus in cellular membranes. The results were further validated by a quantitative real-time PCR analysis. The fine-mapping of a novel QTL for blast resistance by integrative BSA mapping and transcriptome sequencing enhanced the genetic understanding of the mechanism of blast resistance in upland rice. The most suitable genotypes with resistance alleles would be useful genetic resources in rice blast resistance breeding.

## Teaser

Rice blast caused by *Magnaporthe oryzae*is the most devastating disease in rice. Identification of new sources of resistance and the introgression of major resistance genes into elite cultivars are required for sustainable rice production. Taking advantage of an upland rice genotype ‘UR0803’ that exhibits blast resistance, we detected a novel major QTL and four candidate genes for blast resistance in rice by combining genetic mapping and gene expression approaches. Several hybrids highly resistant to blast disease were generated in this study and would be useful genetic resources in rice blast resistance breeding.

## Introduction

Rice (*Oryza sativa*) is a staple and the most important food crop, feeding more than half of the world’s population (Fang *et al.* 2019; Kalia and Rathour 2019). It is grown in both upland and lowland areas of many countries in Asia, Europe and Africa (Fongfon *et al.* 2021). China is among the top producers and consumers of rice grains. Here, the upland rice has a significant contribution to total rice production and also plays an important role in crop rotation in the South to northern areas of the country (Fongfon *et al.* 2021). It provides various economic benefits regarding fertilizers in rotation with potato, wheat and maize in various parts of the country. The productivity of upland rice is often limited by various constraints, including biotic and abiotic stresses (Chumpol *et al.* 2018). Rice blast caused by *Pyricularia grisea* (synonymous *Pyricularia oryzae*; teleomorph *Magnaporthe grisea*) is considered one of the most devastating biotic stresses due to its destructive influences and extensive distribution in various vegetative parts of the plant throughout the crop life cycle. During the vegetative growth stage, it is typically characterized by spindle-shaped lesions on the leaf blade and necrotic lesions at leaf collar (Kalia and Rathour 2019). It spreads quickly in rainy seasons and can cause up to 80 % yield loss in favourable conditions (Chumpol *et al.* 2018; Wangsawang *et al.* 2019; Kumar *et al.* 2021). Therefore, searching for resistant genetic resources of rice and developing control strategies against the continuously evolving pathogens of leaf blast are key research topics in rice.

The development and use of genetically resistant rice varieties are the most economical and effective way to control the disease (Chumpol *et al.* 2018; Fongfon *et al.* 2021). Exploration and exploitation of effective sources of resistance genes and their fine-mapping in the rice genome is a basic step towards effective manipulation of these genes in resistance breeding programmes. Since the first genetic study for rice blast in 1922 (Sasaki 1922), 118 genes have been mapped and 28 genes have been cloned (Ashkani *et al.* 2016; Fang *et al.* 2019; Kalia and Rathour 2019). A significant chromosome-specific linkage for resistance genes has been reported on chromosomes 6, 11 and 12 (Ning *et al.* 2020). The major clusters or quantitative trait loci (QTLs) for leaf blast resistance were observed on chromosomes 6, 11 and 12 of rice harbouring 21, 27 and 27 genes, respectively, while the minimum number of resistance genes (one gene on each chromosome) was observed on chromosome 3 and 7 (Chen *et al.* 2016; Fang *et al.* 2019; Kalia and Rathour 2019; Meng *et al.* 2021; Mishra *et al.* 2021). The first successful tagging of blast resistance genes was reported for *Pi-b* (Miyamoto *et al.* 1996). The *Pi2* cluster on chromosome 6 has been reported for the four successfully cloned genes (*Pi2*, *Pi9*, *Pi-gm* and *Piz-t*) (Fang *et al.* 2019; Kalia and Rathour 2019; Meng *et al.* 2021; Mishra *et al.* 2021). Among the 27 mapped genes on chromosome 11, six genes (*Pik*, *Pik-h*, *Pik-m*, *Pik-p*, *Pi1* and *Pi-ke*) were located in Pik cluster (Wu *et al.* 2012; Chen *et al.* 2015; Zhang *et al.* 2016; Fang *et al.* 2019; Meng *et al.* 2021; Mishra *et al.* 2021). The functional evaluation of the majority of cloned resistance genes revealed their role to encode the nucleotide-binding site–leucine rich repeat proteins (Fang *et al.* 2019). The *Pi-d2* was reported to encode a B-lectin receptor kinase (Kouzai *et al.* 2013), while the recessive gene *pi21* encodes a proline-rich protein (Fang *et al.* 2019), *Bsr-d1* encodes a C_2_H_2_-type transcription factor protein (Li *et al.* 2017), and *Bsr-k1* encodes a tetratricopeptide repeats-containing protein (Zhou *et al.* 2018). Up till now, the most economic and effective way to control leaf blast disease is introducing and pyramiding the resistance genes into susceptible elite cultivars (Fang *et al.* 2019; Kalia and Rathour 2019). Various resistance genes (*Pi1*, *Pi5*, *Piz-5*, *Pita* and *Pi-gm*) have been introgressed into elite cultivars by the marker-assisted selection method (Deng *et al.* 2017; Kumar *et al.* 2021). However, few genes were effective in the control of leaf blast resistance specifically in upland rice. Hence, the identification of novel QTL/gene(s) is required for broader spectrum blast resistance in rice.

Bulked segregant analysis (BSA) (Liang *et al.* 2014) is a cluster separation analysis which refer to the use of two parents with contrasting target traits to construct families and the selection of offspring with parental phenotypes. Bulked segregant analysis has been used as an alternative approach to genome-wide association analysis for complex traits in segregating populations (Ehrenreich *et al.* 2010). It has been proven an easy and efficient technique to reveal large-effect QTLs and/or genes for quantitative traits (Liu *et al.* 2012).

Transcriptome sequencing includes the entire repertoire of transcripts in a species, representing functionally expressed biological information in the genome with respect to target traits (Rani and Sharma 2017). With the rapid development of massively parallel sequencing or next-generation sequencing and the maturation of analytical tools during the last few years, the whole-transcriptome analyses to reveal the differentially expressed genes (DEGs) have become a significant tool to evaluate the contrasting traits (Wolf 2013; Chase *et al.* 2021; Ghimire *et al.* 2021). It is an important tool to pinpoint candidate genes involved in important agronomic traits.

The objective of this study was to detect new QTLs and candidate genes for blast resistance using the upland rice genotype UR0803 as a novel source of blast resistance. We mapped the leaf blast resistance QTL and candidate genes in UR0803, and further fine-mapped using the BSA approach and characterized the candidate genes by transcriptome analysis. The study will be helpful and will be a reference for the utilization of this genotype and the candidate genes in rice breeding for blast resistance.

## Materials and Methods

### Plant materials and growth

An upland rice blast-resistant cultivar UR0803 and -susceptible rice variety Lijiang Xintuan Heigu (LTH) (Singh *et al.* 2014) were acquired from Guizhou Institute of plant protection, China. Lijiang Xintuan Heigu is a very popular rice genotype in China (Singh *et al.* 2014) while UR0803 is a local farm rice variety resource collected in Youmai Village, Youmai Township, Wangmo County, Qianxinan Prefecture, Guizhou Province in 2008 (longitude 105.59.879ʹ, latitude 25.03.353ʹ, altitude 738 m). It is locally called precocious dry waxy. To explore the resistance potential and the QTL/gene(s) related to blast resistance in UR0803, the F_1_, F_2_ and BC_1_F_1_ segregating populations were designed from the cross of UR0803 and LTH. The F_2_ population was subjected to QTL mapping for blast resistance. The parental genotypes, F_1_ and BC_1_F_1_ hybrids, and the F_2_ mapping population were grown at Guizhou Institute of plant protection experiment station (Jinzhu Town, Xiaohe District, Guiyang City, Guizhou Province, China) in 2019, 2020 and 2021. Each time, the experiment was laid out in the randomized complete block design (RCBD) with three replications. The plants were grown in two rows per plot. The parent genotypes were grown adjacent to the plots, as resistant and susceptible controls, respectively. At the three leaves stage, the leaves of these plants were inoculated with *Magnaporthe oryzae* for evaluation of blast resistance.

### Inoculation and resistance evaluation

The Guizhou *M. grisea* compulsory strain 07-91-1 from Guizhou, China was used for inoculation in this study, which was provided by Guizhou Institute of plant protection, China. The F_2_ individuals and two parents were inoculated with the pathogen by a spray method as previously described (Fang *et al.* 2019). Three leaves per plant and five plants for each line were inoculated with conidial suspension (3 × 10^4^ conidia per mL). Disease severity rate (for leaf blast) was evaluated based on a visual assessment of disease severity employing the standards of the International Rice Research Institute (score from 0 to 9, where 0 = no lesion; 1 = small brown specks; 3 = small necrotic brown spots, about 1–2 mm; 5 = infection of 4–10 % of the leaf area; 7 = infection of 26–50 % of the leaf area; and 9 = infection of greater than 51 % of the leaf area, in which many leaves are dead) (Chumpol *et al.* 2018).

### Statistical analysis

The analyses of genotypic and environmental variances in accordance with RCBD were performed in SPSS v19. Three years were considered to evaluate the environmental effect. The frequency distribution among the resistant and susceptible plants was estimated and drawn in Microsoft Excel 2010. The observed and expected Mendelian ratio for frequency distribution was validated by chi-square test at the threshold of 0.99 confidence of interval.

### BSA for QTL mapping

**Figure 1.**
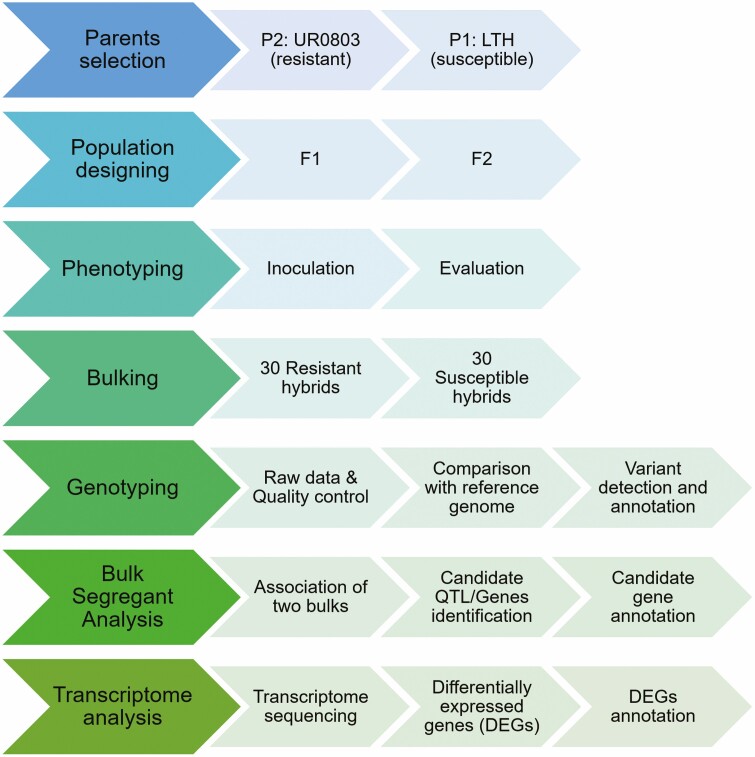
Flow chart diagram of scheme for bulk sergeant analysis to explore the upland rice UR0803 as a resistance source for rice blast.

The DNA pools of 30 disease-resistant and 30 -susceptible individuals of the F_2_ generation **[see****Supporting Information—Table S1****]** were constructed, respectively. In order to further locate the rice blast resistance genes, a high-throughput BSA sequencing was performed and the SNP-index algorithm (Üstünkar *et al.* 2012) was used to obtain the candidate region related to disease resistance in upland rice (Fig. 1).

#### Bulked DNA sequencing and evaluation.

##### Sample detection.

In order to ensure the quality of the DNA library, the genomic DNA was detected and evaluated with three threshold criteria. (i) Agarose gel electrophoresis detection showed the complete and clear main band of genomic DNA, and there was no degradation and RNA contamination. (ii) The ratio of optical density (OD) 260/280 detected by Nanodrop ranged from 1.8 to 2.2, and there was no contamination of protein and visible impurities. (iii) The detection concentration of Qubit 3.0 was greater than 20 ng μL^−1^, and the total amount was more than 2 μg.

##### Library construction.

The purified genomic DNA of the samples was used to construct the library as per the protocol developed for the high-throughput SNP typing method. It was performed in the four major steps; (i) designing the target site-specific primers, and mixing the primers according to the site information for mapping; (ii) taking genomic DNA as a template, and using KAPA2G Fast Multiplex Mix to amplify the target site; (iii) adding the sequencing adapters by secondary PCR; and finally (iv) pooling all the PCR products and purified them with AMPure XP Beads.

##### Library quality control and on-machine sequencing.

The constructed libraries were further used for the quality assessment and sequencing. The quality standards were obtained by: (i) using Qubit3.0 for preliminary quantification; (ii) using Agilent 2100 to detect the insert size of the library with no linker contamination; (iii) using the German ANALYTIKJENA (Jena) QTOWER real-time fluorescent quantitative PCR instrument to accurately quantify the effective concentration of the library, the effective concentration threshold was maintained for >2 nM. The library was pooled according to the target offline data volume, and paired-end 150-bp (PE150) sequencing was performed on the Illumina HiSeq platform.

#### Genotyping, sequencing data and bulk segregant analyses.

##### Cleaning raw data and extraction of SNP and InDel markers.

Raw sequencing data were subjected to quality enhancement by script-based (using C Script) analysis. The reads with (i) more than 10 % of N bases (undetermined bases), (ii) base quality value less than 5 in a read that accounts for more than 50 % of the bases, (iii) more than 10 linker sequence bases, allowing base mismatch rate to be within 10 % and/or (iv) redundant sequences caused by PCR in the process of library construction were removed to get the clean reads for further analyses. The BWA software (Li and Durbin 2010) was used to compare for mapping and alignment of the sequencing data with the reference genome, and SAMTOOL (Danecek *et al.* 2021) was used to convert the comparison results into a BAM file. The mutation (InDels) and variants (SNPs) detection and markers filtration from all samples were performed by GATK (version 4.1.4) software (McKenna *et al.* 2010). The ANNOVAR software (Wang *et al.* 2010) was used to analyse the SNP according to the ‘gff’ file of the reference genome, and InDel to get the VCF file. The polymorphic markers among two parental genotypes were then identified, and their index value of paternal alleles in the two mixed (BSA) pools was calculated. The sliding window processing on the markers of the whole gene was performed by default strategy of 1 Mb to 10 kb to exclude individual false-positive points. The average index value of the selected markers was estimated and used to calculate the ΔSNP index between the two mixed pools to locate the candidate QTL.

##### Fine-mapping and prediction of candidate genes.

A high-throughput SNP typing technique was performed for fine-mapping of disease resistance genes in the candidate QTL region. Eighteen SNP sites evenly distributed in the candidate region were selected, and their amplification primers were designed by Primer3 software using standard parameters (Table 1). Meanwhile, the ePCR program (Iacumin *et al.* 2020) was used to detect the amplification specificity of these primers to ensure that each pair of primers only amplifies the target site. Fourteen selected SNP markers were used to genotype and screen all of the 955 individual plants in the F_2_ population for identification of recombinant events. The genotypic data and the recombinant genotypes were further evaluated for exact estimation of the candidate gene region. The selected region was mapped against the Rice Annotation Project Database (https://rapdb.dna.affrc.go.jp/) to find the candidate genes and their annotations.

**Table 1.** The SNP markers and their primers used to shorten the candidate QTL region on chromosome 11 for rice blast resistance.

	Name	Position	Reference allele	Alternate allele	Forward primer	Reverse primer	Product size	ePCR product details
1	V000652	27 594 376	A	G	TGAAGCAGTTCAATCCCCTTGT	GCAGTAGATGTCACAACAACTCA	76	Chr11_+_27594353_27594428_0_0_76
2	V000943	27 643 915	G	A	CTGAGTCCTAAATGATTCCTCC	TCCAGTGTGTGTTTGCTATT	90	Chr11_+_27643893_27643982_0_0_90
3	V001248	27 671 082	G	A	TCCGCTATCATCAACAGC	AGGATGCCATGAAGTACCT	80	Chr11_+_27671064_27671143_0_0_80
4	V001856	27 705 656	A	G	AGCAGCTCAGAGTATAGCCA	GGCCATTACAGCTTTGCACA	79	Chr11_+_27705625_27705703_0_0_79
5	V003234	27 823 925	G	A	TCATGGCCTCGAAAGGTTGA	TCCTCTTCTCTGTCTTAAGATGT	70	Chr11_+_27823884_27823953_0_0_70
6	V004520	27 996 638	T	C	ATGGCACTCAAAAAGGCCTC	TGACACAAGGTTATCGATTT	76	Chr11_+_27996609_27996684_0_0_76
7	V008885	28 485 963	G	A	TACATCTACCAGCTGCCCAT	TTGTATGTCCTTCCAGGGTAGC	77	Chr11_+_28485918_28485994_0_0_77
8	V009560	28 640 344	T	C	TGCCAACTACGGTATAGGTTGC	CGCAAGGAAGGGTATAAAATCCT	76	Chr11_+_28640298_28640373_0_0_76
9	V010425	28 715 509	T	G	AGCAACATCCACTGGAAGCT	TCTCATCATGTCTCAACCTCACA	75	Chr11_+_28715458_28715532_0_0_75
10	V010563	28 743 785	C	T	TTTAGTAAAGTGCCCGCGCA	GCATGTCCACTAAATAGTCATCA	80	Chr11_+_28743761_28743840_0_0_80
11	V011893	28 824 438	A	C	CATCTGTAGATGTTGGCAAAC	TGCCTATTCATTGTGAAGTCA	79	Chr11_+_28824417_28824495_0_0_79
12	V012072	28 847 850	G	A	AATTCAAGAGCAATTCTACA	ACCTCATGGTACCTAGGTACTGT	108	Chr11_+_28847822_28847929_0_0_108
13	V012234	28 867 805	T	C	ATGACTGTCTTGCGATTCAA	CACGCATCGAATTCAGGTTCA	74	Chr11_+_28867768_28867841_0_0_74
14	V012491	28 894 574	G	A	TGCGCGACGATTCAGTGATA	TCCCCAAAAATATCTGAGATG	72	Chr11_+_28894524_28894595_0_0_72
15	V012708	28 936 971	G	A	TTCTCCTAGCTAGCTTGTCCCT	ACTCGACCTTACAAGTGTCGT	78	Chr11_+_28936918_28936995_0_0_78
16	V012878	28 980 529	G	T	ACACATAAACTTTCAACGTTTTC	GTGTATTTAGTTCACACCAA	78	Chr11_+_28980498_28980575_0_0_78
17	V013013	29 000 230	A	G	AGCCCTGCACAATGACAA	CTCGTTTTTAAGGCAGTGGGT	71	Chr11_+_29000209_29000279_0_0_71
18	V013232	29 011 816	A	G	TGCACGTAACTTGTGACTGGA	AAGTGACGACATGCATGGCT	70	Chr11_+_29011772_29011841_0_0_70

### Transcriptome analysis

The RNA samples from blast-resistant genotype UR0803 and -susceptible genotype LTH were collected at different growth stages after inoculation and the expression analysis was performed to identify the DEGs.

#### RNA extraction and transcriptome sequencing.

The leaf samples were collected from disease-resistant (UR0803) and -susceptible (LTH) parents at 0, 24, 48 and 72 h after inoculation for RNA sequencing. The total RNA was extracted by RNAsimple Total RNA Kit (Tiangene, China). The step-by-step process including, the total RNA quantification, mRNA enrichment, double-strand cDNA synthesis, end repair, splice selection, quality assessment for the PCR amplification library and sequencing was performed at MajorBio Company Limited, Shanghai, China.

#### Expression evaluation and identification of DEGs.

The bowtie2 v 2.3.4 program (Langmead and Salzberg 2012) with zero mismatch parameter was used to compare the reads to the assembled transcriptome. The results were compared with RSEM 2 (v 1.3.1) (Li and Dewey 2011). The number of reads counts on each gene was obtained from each sample and the gene expression level was estimated by the reads per kilobase of transcript per million mapped reads (RPKM) method. The differential gene expression was analysed by comparing read count data from the two subspecies using the DESeq2 program (Anders and Huber 2010) with the *Q*-value < 0.05 and log_2_ fold change > 1 as a threshold.

#### Functional annotation of DEGs.

The functional annotation of genes was performed by mapping the genes to the Kyoto Encyclopaedia of Genes and Genomes (KEGG) (Kanehisa *et al.* 2021), and gene ontology (GO) (Ashburner *et al.* 2000; Camon *et al.* 2004) databases. The results from DEG analysis, candidate region analysis and the functional annotation evaluation were visualized by Microsoft Office (Excel and PowerPoint) 2010 and the R-program v 4.2.0.

### Expression validation by quantitative real-time PCR

The DEGs were further evaluated by qRT–PCR. Total RNAs were extracted from fresh leaf samples with the Easy Spin RNA kit (Aidlab, Beijing, China). The quality of RNA samples was assessed by 1 % agarose gel electrophoresis, while the quantity or concentration of RNA was estimated as per A260/A280 wavelength absorption ratio on the ultraviolet spectrophotometer measurement. The 1.5 μg of RNA was reverse-transcribed by Superscript III kit (Invitrogen, Carlsbad, CA, USA) for cDNA synthesis. The quantitative real-time PCR was performed using SYBR green mixture on an ABI 7500 real-time PCR detection system following the descriptions of Dossa *et al*. (Komivi *et al.* 2018). Each 20 μL reaction mixture included 10 μL of 2× SYBR qPCR Master Mix, 6 μL of nuclease-free water, 2 μL of primer (10 mM) and 2 μL of 4-fold diluted cDNA. The cDNA evaluation was triplicated. The denaturation at 95 °C for 30 s, followed by 40 cycles of 95 °C for10 s, and then annealing and synthesis at 60 °C for 30 s was set as reaction profile. The *Ubiquitin* gene was used as an internal control for normalization. The previously reported method developed by Livak and Schmittgen (2001) was employed for the data analysis.

## Results

### Characterization of resistance to leaf blast in rice

**Figure 2.**
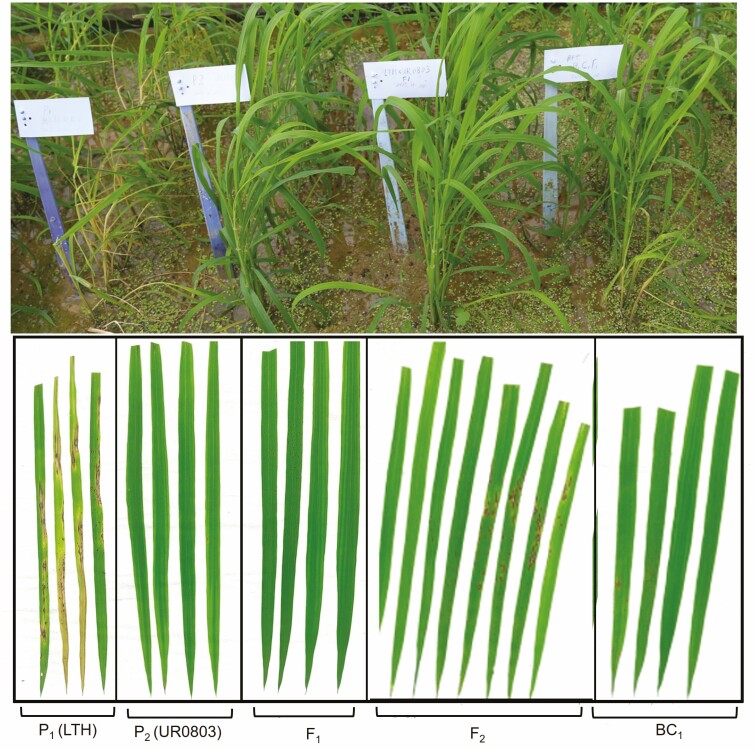
Phenotypic evaluation of susceptible Parent P1 (LTH), resistant Parent P2 (UR0803), their F_1_, F_2_ and BC_1_F_1_ populations.

The upland rice cultivar UR0803 resistant to rice blast was used as a donor for disease resistance genes and crossed to the susceptible rice variety Lijiang Xintuan Heigu (LTH) to develop a mapping population. All the F_1_ individuals were resistant to blast; therefore, the F_2_ segregating population was generated. The characterization for blast resistance in parental genotypes, F_1_ hybrids and the F_2_ population was performed by inoculation with blast compulsory strain 07-91-1 at the three leaves stage. The UR0803 was clearly observable as a resistant genotype in all three replicates (Fig. 2); while among the 160 individuals in the F_2_ population 124 were resistant and 36 were susceptible indicating a clear 3:1 frequency distribution (Table 2). The frequency distribution ratio was non-significantly different from the Mendelian monohybrid F_2_ segregation ratio which clearly indicated the availability of a major gene contributing to the blast resistance in the mapping population.

**Table 2.** Pathogen infestation rate and plants resistance evaluation for parental and hybrid individuals, and segregating populations for rice blast resistance.

Population	Individuals	Resistant (R)	Infected (S)	Disease rate (%)
P_1_ (Lijiang Xintuan Heigu)	17		17	100
P_2_ (UR0803)	21	21		0
F_1_	22	22		0
BC_1_F_1_	20	16	4	20
F_2_	160	124	36	22.5

### BSA and fine QTL mapping of blast resistance by sequencing

**Figure 3.**
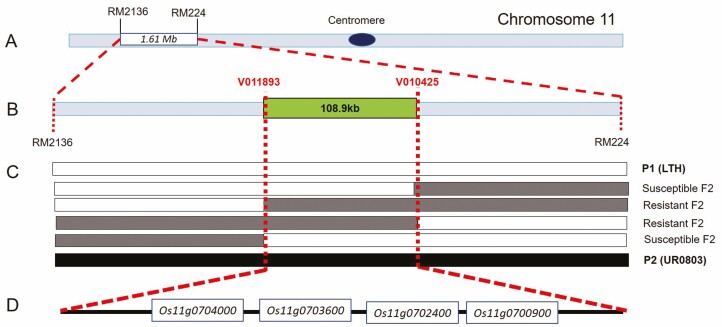
Bulked segregant analysis revealed a blast resistance QTL in upland rice; (A) primary BSA showed a QTL on long arm of the chromosome 11 (the flanking markers are RM2136 and RM224); (B) fine-mapping of candidate QTL by SNP typing (the flanking SNP markers are V011893 and V010425); (C) overlapped mapping analysis of candidate QTL with key recombinants indicating the locus. Empty, lightly filled and dark filled bars represent homozygous fragment from P1 (LTH), heterozygous fragment and homozygous chromosomal fragment from P2 (UR0803), respectively; (D) the four DEGs between two parental genotypes in the QTL region, revealed by transcriptome analysis.

A high-throughput BSA sequencing study was carried out to map the rice blast resistance gene(s). The DNA pools of 30 disease-resistant and 30 -susceptible individuals from the F_2_ generation were constructed **[see****Supporting Information—Table S1****]**, respectively. A total of 50.98 GB of sequencing data was obtained which was reduced to 50.38 GB of cleaned data after filtering. The sequencing depth was 13.6× for parental genotypes while 38.8× for the bulked samples. The average comparison efficiency with the reference genome was 98.52 % with 97.4 % coverage and 26.2× average coverage depth. Finally, a total 1 185 756 SNPs and 135 021 InDels were obtained **[see****Supporting Information—Tables S2****and****S3****]**. A marker density of 1 SNP/363 bp and 1 InDel/3.185 kb of the rice genome was obtained. The computer simulation analysis of SNP index between two sequence pools at a confidence of 0.99 revealed the candidate region in 1.61 Mb ranging from 26 796 502 bp to 28 410 281 bp position on chromosome 11 (Fig. 3; **see****Supporting Information—Fig. S1**). High-throughput BSA sequencing along with SNP-index algorithm yielded a candidate region related to disease resistance within the interval of 27 670 000–29 018 559 bp on chromosome 11, which was right- and left-flanked by the SNP markers RM2136 and RM244, respectively (Fig. 3A). The candidate region contains 110 genes. The region was characterized by 1393 non-synonymous mutant SNPs and 124 InDel sites.

In order to further shorten the candidate genomic interval, a high-throughput SNP genotyping technique based on fine-mapping of disease resistance genes was carried out for all the 955 plants of the F_2_ population. Fourteen SNP markers (Fig. 3B) were developed and used to genotype the mapping population. Finally, the target region was determined between the markers V010425 and V011893 from recombinant individuals (Fig. 3C). The total candidate interval was shortened to 108.9 kb, containing 39 candidate genes **[see****Supporting Information—Table S4****]**.

### Transcriptome evaluation, DEGs and their functional annotation

#### Transcriptome sequencing.

The leaf tissue samples (three biological replicates) from resistant and susceptible parental genotypes were collected for the whole-transcriptome sequencing. The DEG analysis was performed to find the candidate gene(s) in the QTL region. A total of 64.76 million raw reads were obtained with 61.67 million clean reads (Table 3; **see****Supporting Information—Table S2**).

**Table 3.** Data summary for transcriptome assembly of RNA samples collected after 0, 24, 48 and 72 h of inoculation from the upland rice blast-resistant and -susceptible parents.

Samples	Repeat	Q30 (%)	Unique reads (%)	GC (%)	Raw reads	Clean reads	Clean and raw reads ratio (%)	Raw base (Gb)	Clean base (Gb)	Clean and raw base ratio (%)
0-h LTH	1	98.95	47.55	49.5	49 240 246	46 712 896	94.87	7.39	6.85	92.69
	2	99.05	48.05	49	48 281 596	45 769 318	94.8	7.24	6.71	92.68
	3	99.05	45	49.5	56 395 208	53 535 626	94.93	8.46	7.85	92.79
0-h UR0803	1	99.05	49.95	49.5	48 955 216	46 643 676	95.28	7.34	6.82	92.92
	2	99.05	47.65	49.5	52 714 584	50 204 548	95.24	7.91	7.34	92.79
	3	98.95	47.7	49.5	53 531 286	50 672 832	94.66	8.03	7.4	92.15
24-h LTH	1	99	48	50	58 199 992	55 455 778	95.28	8.73	8.11	92.9
	2	99.05	47.1	50	50 672 010	47 919 584	94.57	7.6	7.03	92.5
	3	99.05	45.65	49.5	60 282 596	56 914 450	94.41	9.04	8.34	92.26
24-h UR0803	1	99.05	51.55	49	56 410 656	53 713 286	95.22	8.46	7.83	92.55
	2	98.85	53.25	49.5	45 682 162	43 303 802	94.79	6.85	6.32	92.26
	3	99	51.5	49.5	49 045 986	46 535 696	94.88	7.36	6.79	92.26
48-h LTH	1	99.05	54.15	49.5	48 229 216	45 820 738	95.01	7.23	6.7	92.67
	2	99.05	51.7	49.5	61 317 586	58 218 536	94.95	9.2	8.5	92.39
	3	99.1	53.45	49.5	53 459 344	51 166 006	95.71	8.02	7.49	93.39
48-h UR0803	1	99	51.05	49	44 124 690	41 990 836	95.16	6.62	6.17	93.2
	2	99.05	50.05	49.5	50 339 648	48 082 746	95.52	7.55	7.04	93.25
	3	99	48.75	49.5	53 975 892	51 390 380	95.21	8.1	7.53	92.96
72-h LTH	1	99.05	50.55	49	55 903 836	53 525 032	95.74	8.39	7.85	93.56
	2	99	49.7	49	58 985 338	56 305 662	95.46	8.85	8.24	93.11
	3	98.95	52.1	49.5	50 968 700	48 660 396	95.47	7.65	7.12	93.07
72-h UR0803	1	99.1	46.3	49.5	64 759 242	61 668 734	95.23	9.71	9.05	93.2
	2	99.1	46.7	49.5	63 239 318	60 252 470	95.28	9.49	8.82	92.94
	3	99.1	50.25	49.5	54 466 208	52 034 410	95.54	8.17	7.63	93.39

#### Differential expression and functional enrichment analyses of genes.

**Figure 4.**
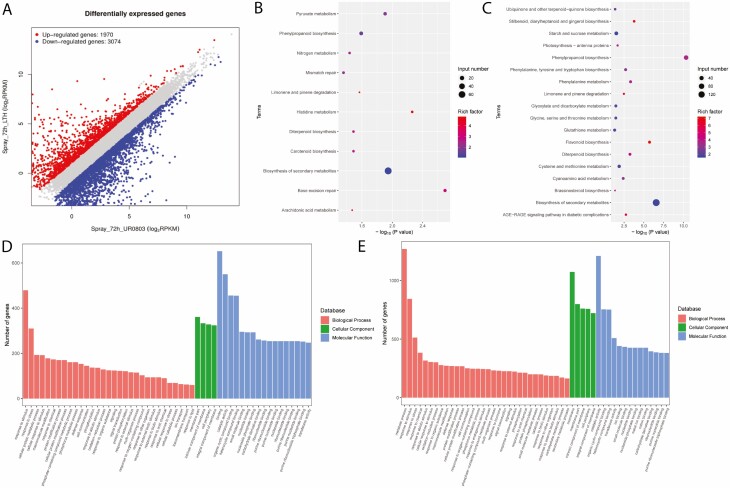
Transcriptome analysis for the samples collected form resistant and susceptible parents at 72 h after inoculation; (A) plotting the DEGs; (B) top enriched KEGG pathways for upregulated and (C) downregulated DEGs; (D) top enriched gene ontological (GO) terms for upregulated and (E) downregulated DEGs.

**Figure 5.**
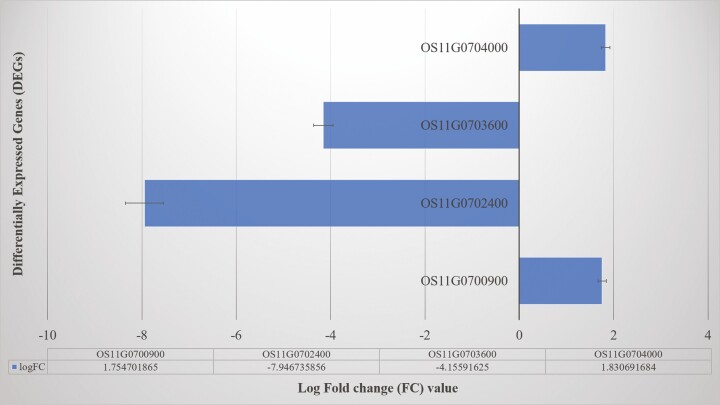
Differentially expressed genes and their log fold change (logFC) values between susceptible (LTH) and resistant (UR0803) rice genotypes.

A non-standard normal distribution was observed for RPKM values-based gene expression level among the samples. There was a total of 5044 genes observed to be differentially expressed in the resistant parent (UR0803) and susceptible parent (LTH) at 72 h after inoculation. Among these DEGs, 423 were on chromosome 11 (Fig. 4; Table 4), out of which four DEGs including two upregulated (*Os11g0700900*, *Os11g0704000*) and two downregulated (*Os11g0702400*, *Os11g0703600*) genes detected as candidate genes in this study. All candidate DEGs showed a signification expression variation with −log (*P*) values ranging from 7.02 to 25.20 (**see****Supporting Information—Table S5**; Fig. 5).

**Table 4.** Differentially expressed genes between susceptible (LTH) and resistant (UR0803) under various treatments including 0, 24, 48 and 72 h after inoculation.

Treatment*	Expression regulation	DEGs in chromosome												
		1	2	3	4	5	6	7	8	9	10	11	12	Total
L0 vs. U0	Up	279	172	163	151	101	123	117	118	99	115	174	97	1709
	Down	509	379	444	350	312	294	257	217	158	207	251	190	3568
	Total	788	551	607	501	413	417	374	335	257	322	425	287	5277
L24 vs. U24	Up	440	310	275	264	182	229	219	181	165	171	212	131	2779
	Down	493	359	382	371	276	280	257	230	164	195	246	184	3437
	Total	933	669	657	635	458	509	476	411	329	366	458	315	6216
L48 vs. U48	Up	587	493	477	357	330	345	316	283	197	228	332	235	4180
	Down	412	309	308	299	199	225	208	174	156	139	214	153	2796
	Total	999	802	785	656	529	570	524	457	353	367	546	388	6976
L72 vs. U72	Up	279	213	180	155	124	170	141	164	94	130	201	119	1970
	Down	467	313	361	296	249	241	242	186	148	159	219	193	3074
	Total	746	526	541	451	373	411	383	350	242	289	420	312	5044
U0 vs. U24	Up	141	83	105	123	72	70	72	44	60	70	88	51	979
	Down	236	156	155	152	96	100	119	94	93	87	122	87	1497
	Total	377	239	260	275	168	170	191	138	153	157	210	138	2476
U0 vs. U48	Up	617	404	474	420	285	338	287	253	237	223	309	210	4057
	Down	851	671	793	548	484	454	441	388	306	296	326	332	5890
	Total	1468	1075	1267	968	769	792	728	641	543	519	635	542	9947
U0 vs. U72	Up	477	315	344	298	223	269	237	200	161	166	243	204	3137
	Down	580	445	524	369	358	313	283	284	188	217	239	220	4020
	Total	1057	760	868	667	581	582	520	484	349	383	482	424	7157

*In treatments ‘L’ stands for LTH, and ‘U’ stands for UR0803; 0, 24, 48 and 72 indicate that the RNA samples collected after 0, 24, 48 and 72 h after inoculation of *Magnaporthe oryzae*.

#### Candidate genes in fine-mapped region and their functional annotation.

Among the identified DEGs, four were located in the mapped QTL region in this study: (i) *Os11g0702400* was downregulated in the sensitive parent after inoculation and annotated for zinc finger, C_2_H_2_-type domain-containing protein involved in response to stimulus, nucleic acid bind, regulation of gene expression and metabolic processes like ontological processes; (ii) *Os11g0703600* was also downregulated in the sensitive parent after inoculation and annotated for conserved hypothetical protein in membrane and membrane-bounded organelles; (iii) *Os11g0700900* was upregulated in the sensitive parent after inoculation and annotated for glycoside hydrolase, catalytic core domain-containing protein functional for responses to various stresses and environmental factors; and (iv) *Os11g0704000* was upregulated in the sensitive parent after inoculation and annotated for seleno-protein (SelT/SelW/SelH) family protein to be expressed in intracellular membrane-bounded organelles **[see****Supporting Information—Tables S4****–****S7****]**.

### Validation of RNA analysis by qRT–PCR

**Figure 6.**
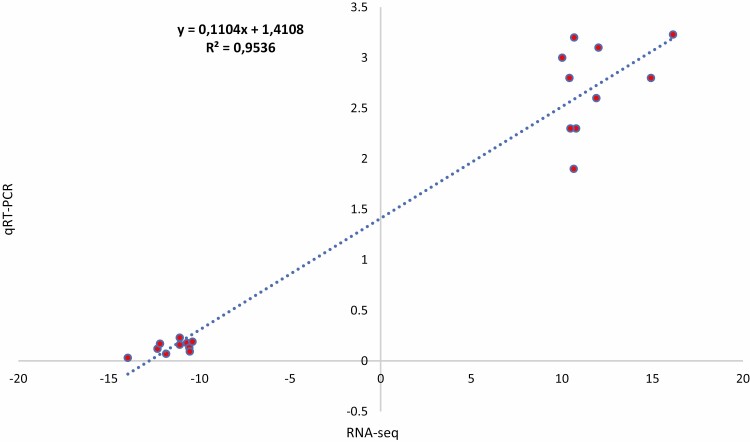
Pearson correlation analysis of the expression profile of the four candidate genes between qRT–PCR and RNA-Seq.

To validate the expression of identified DEGs for blast resistance in the mapped QTL region, all four up-, and downregulated genes were selected and used for qRT–PCR expression profiling. The qRT–PCR results of the selected genes were consistent with that of RNA-Seq analysis (Pearson correlation = 95 %) (Fig. 6). This result supports the DEG analysis and subsequent interpretations.

## Discussion

The development of resistant varieties is one of the most adopted ways to protect crops from biotic stresses. Nevertheless, it is not a durable method as the resistance in elite varieties often diminishes within a few years of being released (Mishra *et al.* 2021). The causal fungus for rice blast *M. oryzae* is continuously evolving due to environmental changes, resulting in natural mutations with a significantly high mutation rate (Mishra *et al.* 2021). Therefore, the induction of blast resistance in rice remains challenging. Although the trait is not durable, the resistance caused by a single major gene may remain effective for many years (Mishra *et al.* 2021). Therefore, it is imperative to understand the regulation of plant defence mechanisms, identification of novel sources of durable resistance as well as determine the functional genes and controlling elements responsible for the effectiveness and breakdown of resistance.

Resistance to blast disease is also known to be host-specific and the identified resistance genes have been reported to be effective against specific strains of *M. oryzae* (Mishra *et al.* 2021). Hence, the traditional rice varieties are known to possess one or two dominant genes (Mishra *et al.* 2021). Historically, the rice genotype PS2 containing at least two dominant genes *Pi-b* and *Pi54* (Rahim *et al.* 2013; Tanweer *et al.* 2015) and IR64 containing *Pi* genes (Windarsih and Utami 2017) have been widely used as a source for blast resistance in the development of high-yielding rice cultivars (Mishra *et al.* 2021). Nonetheless, the genetic resources for blast resistance in upland strains are still less reported. In this study, a blast-resistant upland rice genotype was evaluated. It was used as a donor parent of rice blast resistance allele to develop the mapping population. Thereby, it resulted in the mapping of a major QTL on chromosome 11 followed by fine-mapping of four candidate genes.

Previously, different populations of diverse rice germplasm have been screened with various molecular markers such as SSR, InDel, SNP and gene-specific markers that have been reported for most of the mapped and cloned blast resistance genes. It has been shown that the diverse germplasm had resistant marker alleles (Mishra *et al.* 2021). However, it has been observed that many of these markers are non-functional for blast resistance breeding in rice. It may be due to the lack of tightly linked markers to the candidate genes. To overcome this shortcoming, SNP markers were obtained from the high-throughput sequencing of DNA pools of resistant and susceptible genotypes in this study. The BSA and SNP marker-based genotyping resulted in the identification of a very short QTL region (108 kb) as compared to previous reports on QTL mapping in rice (Wang *et al.* 2016).

The high-throughput sequencing of cDNA obtained from extracted RNA of plant tissues has become an important technique to explore gene expression and the DEGs among various treatments and genotypes (Zhang *et al.* 2021). In this study, the expression analysis revealed four DEGs located in the fine-mapped candidate region. Two of them were upregulated and the other two were downregulated in the susceptible plant after inoculation. None of these genes has been reported previously, indicating the novelty of candidate QTL for leaf blast resistance. The locus is situated close to the previously reported R-gene cluster that has been mapped on the telomeric end of rice chromosome 11 containing at least nine resistance genes of which seven genes were the alleles of a single gene *Pi-k* locus (Hua *et al.* 2012; Singh *et al.* 2015; Fang *et al.* 2019; Fongfon *et al.* 2021; Mishra *et al.* 2021). A similar report has also been found in which a QTL *qPbh-11-1* was mapped in the *Pi-k* gene cluster (Wu *et al.* 2013). A major leaf blast resistance gene *Pi-hk1(t)* has also been identified from the donor genotype Heikezijing (Wu *et al.* 2013). Another gene *Pi-jnw1* conferring the panicle and leaf blast resistance has been identified from a *japonica* landrace Jiangnanwan on chromosome 11 between markers RM27273 and RM27381 (Wang *et al.* 2016).

Among the downregulated candidate genes, *Os11g0702400* was annotated for zinc finger, C_2_H_2_-type domain-containing protein involved in various gene ontological processes including the response to stimulus, nucleic acid bind, regulation of gene expression and metabolic processes. Previously, a gene *Bsr-d1* encoding C_2_H_2_-type transcription factor protein has also been reported to confer broad-spectrum blast resistance in rice (Li *et al.* 2017). Its downregulation could inhibit the degradation of H_2_O_2_ resulting in the enhanced blast resistance in rice (Fang *et al.* 2019). Hence, the available upland germplasm and the resistant hybrid individuals from the F_2_ population used in this study for the QTL/gene mapping may a useful genetic resource for durable blast resistance in upland rice.

## Conclusions

The study aimed at mining QTLs for blast resistance in the upland rice UR0803 as a novel source of blast resistance. Using an integrative approach of BSA, SNP genotyping and transcriptome analysis, we successfully identified a novel candidate QTL and four candidate genes linked to blast resistance in rice. The novel QTL region will be useful for the development of durable resistance to rice blast. The functions of its underlying genes should be analysed to better understand the varying levels of resistance exhibited against the blast isolates. The identification of blast resistance genes in this study contributes to the genetic understanding of disease resistance and will help for marker-assisted breeding against blast resistance in upland rice.

## Supporting Information

The following additional information is available in the online version of this article—

**Table S1.** List of parental genotypes along with 30 most resistant and 30 most susceptible individuals from F_2_ mapping population for bulked segregant analysis.

**Table S2.** Summary of sequencing data for bulked segreant analysis (BSA) from the blast-resistant and -susceptible DNA pools obtained from upland rice mapping populations.

**Table S3.** Characterization descriptive in candidate region of quantitative trait locus (QTL) mapping by comparison of sequences from resistant and susceptible parents.

**Table S4.** List of 39 candidate genes and their functional annotations in mapped quantitative trait locus (QTL) region.

**Table S5.** List of 423 differentially expressed genes between resistant and susceptible parents on chromosome 11.

**Table S6.** Functional annotation of four candidate genes in gene ontology and Kyoto Encyclopaedia of Genes and Genomes (KEGG) databases.

**Table S7.** The genomic variants, their alternate alleles, and physical position in candidate genes.

**Figure S1.** Chromosome-wide SNP index estimated by two sequence pools; index1 indicates susceptible pool; index2 indicates resistant pool; while Delta indicates the variation of SNP index between two pools.

plac047_suppl_Supplementary_Figure_S1

plac047_suppl_Supplementary_Tables

## Data Availability

The raw sequencing data have been submitted to NCBI SRA under the project numbers: PRJNA754205 (transcriptome) and PRJNA795286 (BSA).

## Notes

**Phenome, Genome & Environment. Chief Editor:** Colleen Doherty
