# Supplementary figures and images for: Integrated genetic analysis of leaf blast resistance in upland rice: QTL mapping, bulked segregant analysis and transcriptome sequencing
Source: AoB Plants. 2022 Oct 14;14(6):plac047. doi: 10.1093/aobpla/plac047 (PMC9773827; doi:10.1093/aobpla/plac047)

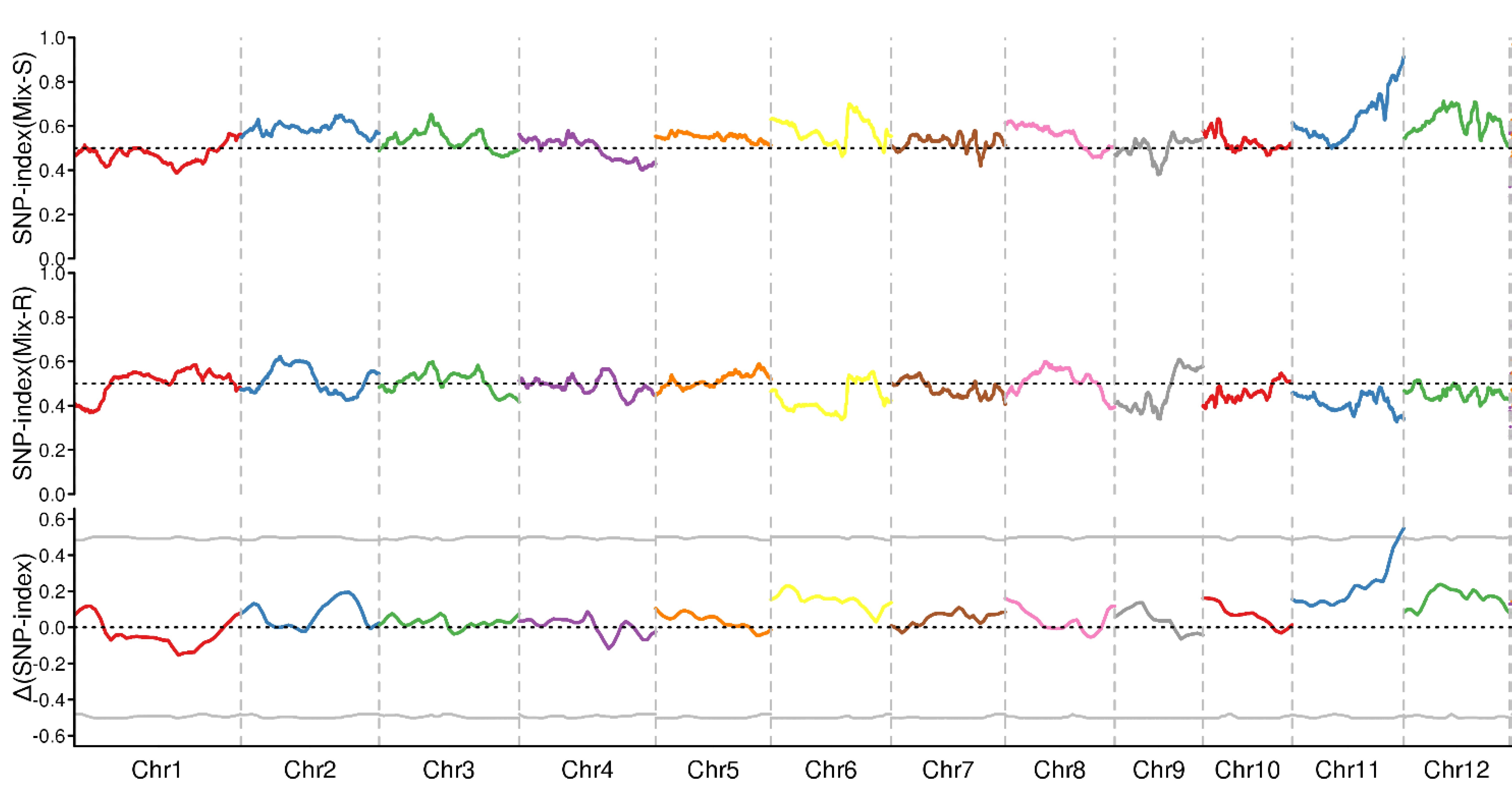

Supplement: plac047_suppl_Supplementary_Figure_S1 [file plac047_suppl_supplementary_figure_s1.jpeg]
